# A critical review of the role of Fc gamma receptor polymorphisms in the response to monoclonal antibodies in cancer

**DOI:** 10.1186/1756-8722-6-1

**Published:** 2013-01-04

**Authors:** James D Mellor, Michael P Brown, Helen R Irving, John R Zalcberg, Alexander Dobrovic

**Affiliations:** 1 Pharmacy Department, Peter MacCallum Cancer Centre, St Andrew’s Place, East Melbourne, Victoria, 3002, Australia; 2 Monash Institute of Pharmaceutical Sciences, Monash University, Parkville, Victoria, 3052, Australia; 3 Molecular Pathology Research and Development Laboratory, Peter MacCallum Cancer Centre, St Andrew’s Place, East Melbourne, Victoria, 3002, Australia; 4 Cancer Clinical Trials Unit, Royal Adelaide Hospital, North Terrace, Adelaide, South Australia, 5000, Australia; 5 Division of Cancer Medicine, Peter MacCallum Cancer Centre, St Andrew’s Place, East Melbourne, Victoria, 3002, Australia; 6 Sir Peter MacCallum Department of Oncology, University of Melbourne, Parkville, Victoria, 3010, Australia; 7 Department of Pathology, University of Melbourne, Parkville, Victoria, 3010, Australia; 8 Pharmacy Department, Peter MacCallum Cancer Centre, Locked Bag 1, A’Beckett Street, East Melbourne, Victoria, 8006, Australia

**Keywords:** FCGR2A, FCGR3A, trastuzumab, rituximab, cetuximab, ADCC

## Abstract

Antibody-dependent cellular cytotoxicity (ADCC) is a major mechanism of action of therapeutic monoclonal antibodies (mAbs) such as cetuximab, rituximab and trastuzumab. Fc gamma receptors (FcgR) on human white blood cells are an integral part of the ADCC pathway. Differential response to therapeutic mAbs has been reported to correlate with specific polymorphisms in two of these genes: *FCGR2A* (H131R) and *FCGR3A* (V158F). These polymorphisms are associated with differential affinity of the receptors for mAbs. This review critically examines the current evidence for genotyping the corresponding single nucleotide polymorphisms (SNPs) to predict response to mAbs in patients with cancer.

## Targeted therapy utilising monoclonal antibodies

Advances in our understanding of the molecular processes involved in carcinogenesis have allowed the development of drugs that target specific cellular processes in malignant cells. The use of such targeted therapies has become widespread in oncology. Due to this targeted approach, many of the ubiquitous side-effects of conventional chemotherapy, such as myelosuppression, are reduced or eliminated.

Monoclonal antibodies (mAbs) are an important group of targeted therapies which are directed against transmembrane proteins with extracellular domains. Several mAbs have entered routine clinical practice: notable examples include trastuzumab (Herceptin®), rituximab (Mabthera®/Rituxan®) and cetuximab (Erbitux®) (Table 1). Trastuzumab targets the human epidermal growth factor receptor family member HER2 and is indicated as the standard of care in HER2 over-expressing breast and gastric cancers. Rituximab targets CD20 receptors expressed on most malignant B cells and is used as the standard treatment for B-cell malignancies such as non-Hodgkin’s lymphoma (NHL) and chronic lymphocytic leukaemia (CLL). Cetuximab targets the epidermal growth factor receptor (EGFR) and is used in *KRAS*-wild type, metastatic colorectal cancer.

**Table 1:** Summary of therapeutic mAbs included in this review

**Generic name**	**Brand name**	**Indication**	**Construct**	**Isotype**	**Target**
rituximab	MabThera®/ Rituxan®	CD20^+^ lymphoma and CLL	chimeric	IgG1	CD20
trastuzumab	Herceptin®	breast cancer; gastric cancer	humanized	IgG1	HER2
cetuximab	Erbitux®	colorectal cancer; head and neck cancer	chimeric	IgG1	EGFR

It is desirable to avoid exposing patients to a costly and potentially toxic treatment if they have a reduced chance of response. Cancer patients are thus often tested for specific biological markers (biomarkers) that have been found in clinical trials to be predictive of response to targeted agents. A typical example is *HER2* amplification which is used to select patients for trastuzumab treatment. Another example is mutation of codon 12 of the *KRAS* gene which identifies patients who are unlikely to respond to cetuximab. While targeted agents do not have the same side effect profile as conventional chemotherapy, they do still cause side effects, some of which can be severe [1].

The problem is that not all of those patients who are predicted to respond will do so, even if the biomarker predicts response. For example, only 25-30% of HER2 amplification-positive metastatic breast cancer patients will respond to trastuzumab [2]. Therefore, there is a need to identify and validate additional robust biomarkers of response to therapy in cancer patients. Understanding the mechanisms of action of mAbs is of critical importance.

### Antibody-dependent cellular cytotoxicity and Fc gamma receptors

**Figure 1:**
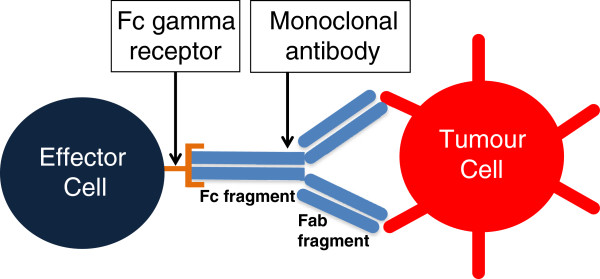
The antibody-dependent cellular cytotoxicity complex.

Antibody-dependent cellular cytotoxicity (ADCC) has been identified pre-clinically as an important mechanism in the elimination of tumour cells. ADCC depends on the bifunctional structure of immunoglobulin G (IgG) molecules. Therapeutic mAbs are typically molecules of the IgG class and comprise an antigen-binding fragment (Fab) that engages the tumour cell antigen and a crystalline fragment (Fc) that binds a Fc gamma receptor (FcgR) on an effector cell such as a natural killer (NK) cell, monocyte, or macrophage (see Figure 1).

ADCC is initiated when the Fab and Fc portions of the mAb engage both tumour cell antigen and an activating FcgR, respectively, thus creating a bridge from the tumour cell to the effector cell. Target cell recognition is then coupled to a lytic attack on the target cell mounted by effector cells [3,4]. The importance of this interaction is demonstrated by the lower anti-tumour activity of mAbs in FcgR-deficient mice compared to wild-type mice [5]. ADCC is considered to be a major mode of action of many therapeutic mAbs, including treatments for cancer [5-8].

There are three classes of FcgRs based on genetic homology (*FcgR1*/CD64, *FcgR2*/CD32 and *FcgR3*/CD16) [9,10]. Each type is encoded by specific genes located in the same region of the long arm of chromosome 1. There are several different closely related genes for each FcgR which have different cell type-specific patterns of expression [9]: *FCGR1A, FCGR1B, FCGR1C; FCGR2A*, *FCGR2B1*, *FCGR2B2*, *FCGR2B3*, *FCGR2C*; *FCGR3A* and *FCGR3B*.

Even though the importance of ADCC is recognised [11], other FcgR-mediated processes contribute to adaptive cellular immunity. While NK cells express FcgR3a rather than FcgR2a, antigen presenting cells such as dendritic cells, macrophages and B cells express both FcgR2a and FcgR3a. Antigen presenting cells use FcgR-mediated endocytosis of immune complexes and phagocytosis of antibody-coated tumour cells as efficient means of tumour antigen processing and presentation, which can result in tumour-directed T-cell immunity [12]. The clinical significance of ‘vaccine-like’, and presumably FcgR-mediated, effects of therapeutic mAbs is increasingly recognised [13,14].

### FcgR polymorphisms

Single nucleotide polymorphisms (SNPs) are single base changes in a gene that occur at a significant frequency (often defined as more than 1%) in a population. SNPs in the coding region of a gene often result in amino acid changes that may alter the functioning of the affected proteins. Certain SNPs in the coding regions of the *FCGR2A* and *FCGR3A* genes appear to have clinical significance as they have been reported to correlate with responses to therapeutic mAbs and these form the principal subject of this review.

A coding polymorphism in the extracellular domain of *FCGR2A* has been described where a C> T substitution (denoted as rs1801274) changes the amino acid at position 131 from histidine to arginine [15]. This polymorphism is conveniently described by its amino acid change His131Arg (H131R using the one letter amino acid nomenclature). The *FCGR2A* receptor binds to different classes of IgGs, with highest affinity for human IgG1 and IgG3 [2]. Position 131 is polymorphic for binding of human IgG2 but not of human IgG1, with the H131 allelic form of FcgR2a seeming to be the only class of FcgR that interacts well with IgG2 [15].

A second important FcgR coding polymorphism occurs in extracellular domain 2 of *FCGR3A.* A T> G substitution changes valine to phenylalanine at position 158 (Val158Phe or V158F) [16,17]. This polymorphism (rs396991) is occasionally denoted in the literature as V176F [16] (and once as *FCGR3A* 818A> C ! [18]). The residue at position 158 directly interacts with the lower hinge region of IgG1 [19,20].

### Therapeutic activity of monoclonal antibodies reported to be affected by FcgR polymorphisms

While any mAb directed to an extracellular antigen may trigger an ADCC response mAbs of IgG1 isotype invoke the strongest response [21]. An important role for the FcgR phenotype is indicated by the observation that NK cells from donors homozygous for *FCGR3A* 158 V (V/V) bound more IgG1 compared with cells from donors who were homozygous for *FCGR3A* 158 F (F/F) [16,17]. Here, we review pre-clinical and clinical data concerning the effects of FcgR polymorphisms on the activity of some widely used therapeutic mAbs which all belong to the IgG1 isotype.

### Pre-clinical and clinical studies

#### Trastuzumab

Trastuzumab is a humanized anti-HER2 IgG1 mAb effective in treating breast and gastric cancers which overexpress HER2. However, only 25%-30% of patients with metastatic HER2-positive breast cancers will respond to trastuzumab [2] and only 30% of HER2-positive patients treated with neoadjuvant trastuzumab will achieve a complete pathological response [22]. In addition, between 2-5% of patients will suffer from clinical cardiac dysfunction as a side effect of trastuzumab therapy [20]. Thus identifying biomarkers that will predict the response to trastuzumab is desirable. As part of the response to trastuzumab may be due to ADCC [2], FcgR polymorphisms are potential biomarkers of response.

In a pre-clinical study, trastuzumab-mediated ADCC of autologous peripheral blood mononuclear cells (PBMNCs) was measured by a chromium-51 release assay using a *HER2*-positive human breast cancer cell line (MDA-MB-361) as a target. The ADCC analysis showed that PBMNCs of *FCGR2A* H/H and/or *FCGR3A* V/V genotypes caused significantly higher trastuzumab mediated cytotoxicity than PBMNCs of other genotypes [2].

A retrospective, non-randomised study of trastuzumab in 54 patients with HER2-positive metastatic breast cancer found a significant difference in the objective response rate depending on the *FCGR2A* and *FCGR3A* genotypes [2]. Patients were treated with trastuzumab plus a taxane (paclitaxel or docetaxel) with a contemporaneous population of patients receiving single-agent taxane serving as controls. Patients who had *FCGR2A* 131 H/H and/or *FCGR3A* 158 V/V genotypes had a significantly better objective response rate and progression free survival (PFS) with trastuzumab therapy than patients with neither genotype (the PFS estimates were 30.3 and 12.8 months respectively; *p* = 0.01). A multivariate analysis led the authors to conclude that the combination of the two favorable *FCGR2A* and *FCGR3A* genotypes was an independent predictive factor of response [2].

Another retrospective, non-randomised study examined the effect of the FcgR polymorphisms on trastuzumab efficacy in both the neoadjuvant and metastatic HER2-positive breast cancer settings [22]. The neoadjuvant group consisted of 15 patients who received doxorubicin and cyclophosphamide followed by weekly paclitaxel and trastuzumab. Thirty-five patients in the metastatic group received weekly single-agent trastuzumab until disease progression. They reported that the *FCGR2A* 131 H/H genotype significantly correlated with pathological response in the neoadjuvant group (71% for H/H vs 0% for H/R and R/R; *p* = 0.015). The *FCGR3A* 158 V/V genotype was found not to correlate with response. In the metastatic group, a significant difference in objective response rate was observed between the *FCGR2A* 131 H/H patients and the H/R or R/R patients (*p* = 0.043). A non-statistically significant trend was reported in *FCGR3A* 158 V/V patients showing an overall higher response rate than V/F or F/F patients (40% for V/V vs 10% for F/V and F/F *p* = 0.053). The PFS of *FCGR2A* 131 H/H patients was found to be significantly longer than that of H/R or R/R patients (9.2 months vs 3.5 months, *p* = 0.034). In contrast, no statistically significant difference in the PFS of *FCGR3A* 158 V/V patients compared with V/F or F/F patients was observed (8.5 months vs 5.3 months, *p* = 0.37).

The largest study to date examining the effects of FcgR polymorphisms on the response to trastuzumab is that of Hurvitz et al. [23]. The patients were part of the Breast Cancer International Research Group (BCIRG)-006 study of patients receiving adjuvant trastuzumab with chemotherapy for HER2-positive early stage breast cancer. BCIRG-006 was a randomized clinical trial in which two trastuzumab containing experimental arms (both using the same dose of trastuzumab – 8 mg/kg loading does followed by 6 mg/kg every 3 weeks for a total of 12 months) were compared to a non-trastuzumab control arm. Germline DNA from 1218 patients and 1189 patients was genotyped for the H131R and V158F SNPs, respectively. There was no statistically significant difference in disease free survival (DFS) based on FcgR genotypes (*FCGR2A* H/H vs H/R vs R/R, log rank test, *p* = 0.81, and *FCGR3A* V/V vs V/F vs F/F, log rank test, *p* = 0.33). *Interestingly*, in the trastuzumab arms, there was no statistically significant difference in DFS by *FCGR2A* (*p* = 0.76) or *FCGR3A* (*p* = 0.98) genotype. Furthermore, when a sub-analysis of the genotypes previously found to have been favorable by Musolino et al. [2] (*FCGR2A* H/H and *FCGR3A* V/V) compared to the other genotypes was performed, once again there was no statistically significant difference in DFS (*p* = 0.67). Hurvitz et al. also looked at a 53 patient cohort of metastatic breast cancer patients who also did not show a difference in PFS according to FcgR genotype. The authors concluded that *FCGR2A* H131R and *FCGR3A* V158F genotype did not correlate with trastuzumab efficacy in HER2-positive breast cancer.

Although the two small, underpowered, retrospective and non-randomised studies [2,22] show that FcgR polymorphisms are associated with anti-tumour efficacy of trastuzumab, the well powered analysis of Hurvitz et al. [23] indicates that FcgR polymorphisms are not useful as predictive biomarkers of trastuzumab response in HER2-positive breast cancer, particularly in early stage disease. However, the patients analysed by Hurvitz show a major departure from Hardy Weinberg equilibrium for FCGR3A but not for FCGR2A. The FCGR3A heterozygotes were under-represented indicating that there probably was an admixture of multiple ethnicities (Table 2). Moreover, in the subset of adjuvant patients genotyped in the study (N = 1,286), the trastuzumab benefit appeared to be non-statistically significant, unlike that seen in the overall BCIRG-006 trial population (N = 3,222).

**Table 2:** Hardy-Weinberg analysis of studies examining the effect of FcGR3a genotype on outcome

***FCGR3A *genotype**	**Actual frequency of genotypes (n)**	**Expected frequency of genotypes (n)**	**Hardy-Weinberg equilibrium *x***^**2**^**(1 degree of freedom)**	**Genotyping methodology**
	Musolino, 2008 (trastuzumab)			Nested PCR-based allele-specific restriction analysis assay
G/G (V/V)	11	10.67	0.03	
G/T (V/F)	26	26.67		
T/T (F/F)	17	16.67		
	Tamura, 2011 (trastuzumab) (Neoadjuvant group)			Goldengate genotyping
G/G (V/V)	7	6.67	0.15	
G/T (V/F)	6	6.67		
T/T (F/F)	2	1.67		
	Tamura, 2011 (trastuzumab)(Metastatic group)			Goldengate genotyping
G/G (V/V)	15	15.78	0.36	
G/T (V/F)	17	15.44		
T/T (F/F)	3	3.78		
	Hurvitz, 2011 (trastuzumab)			Nested PCR followed by Sanger sequencing (confirmed by MassARRAY)
G/G (V/V)	169	137.61	**16.45**	
G/T (V/F)	471	533.78		
T/T (F/F)	549	517.61		
	Cartron, 2002 (rituximab)			Nested PCR followed by allele-specific restriction enzyme digestion
G/G (V/V)	10	9	0.34	
G/T (V/F)	22	24		
T/T (F/F)	17	16		
	Weng, 2003 (rituximab)			Nested PCR followed by allele-specific restriction enzyme digestion. (confirmed by direct sequencing)
G/G (V/V)	13	12.52	0.05	
G/T (V/F)	40	40.97		
T/T (F/F)	34	33.52		
	Persky, 2012 (rituximab)			TaqMAN SNP Assay
G/G (V/V)	5	7.04	1.45	
G/T (V/F)	29	24.92		
T/T (F/F)	20	22.04		
	Kim, 2006 (rituximab)			Nested PCR followed by allele-specific restriction enzyme digestion
G/G (V/V)	53	56.64	2.74	
G/T (V/F)	54	46.73		
T/T (F/F)	6	9.64		
	Dornan, 2010 (rituximab)			Allele-specific PCR with SYBR Green
G/G (V/V)	49	53.07	1.0	
G/T (V/F)	202	192.6		
T/T (F/F)	168	172.7		
	Ghesquieres, 2012 (rituximab)			TaqMAN SNP Assay with specific fluorescent dye–labeled (FAM and VIC) MGB probes
G/G (V/V)	68	66.96	0.04	
G/T (V/F)	215	217.09		
T/T (F/F)	177	175.96		
	Carlotti, 2007 (rituximab)			PCR with fluorescent labeled probes followed by melt curve analysis
G/G (V/V)	17	17.2	0.01	
G/T (V/F)	46	45.59		
T/T (F/F)	30	30.2		
	Prochazka, 2011 (rituximab)			Nested PCR followed by allele-specific restriction enzyme digestion
G/G (V/V)	7	9.44	1.42	
G/T (V/F)	43	38.11		
T/T (F/F)	36	38.44		
	Bibeau, 2009 (cetuximab)			PCR followed by multiplex allele-specific PCR (SYBR Green fluorescence)
G/G (V/V)	10	14.59	**5.02**	
G/T (V/F)	43	33.82		
T/T (F/F)	15	19.59		
	Etienne-Grimaldi, 2012 (cetuximab)			Nested PCR followed by allele-specific restriction enzyme digestion
G/G (V/V)	6	6.71	0.19	
G/T (V/F)	25	23.58		
T/T (F/F)	20	20.71		
	Zhang, 2007 (cetuximab)			Allele-specific PCR
G/G (V/V)	5	4.11	0.44	
G/T (V/F)	14	15.77		
T/T (F/F)	16	15.11		
	Zhang, 2010 (cetuximab)			PCR – restriction fragment length polymorphism technique
G/G (V/V)	23	17.27	**8.11**	
G/T (V/F)	21	32.47		
T/T (F/F)	21	15.27		
	Dahan, 2011 (cetuximab)			PCR – restriction fragment length polymorphism technique
G/G (V/V)	6	4.57	0.88	
G/T (V/F)	20	22.86		
T/T (F/F)	30	28.57		
	Paez, 2010 (cetuximab)			48.48 dynamic array (BioMark system)
G/G (V/V)	16	12.81	1.89	
G/T (V/F)	41	47.38		
T/T (F/F)	47	43.81		

### Rituximab

A number of antibodies have been developed against the CD20 antigen which is widely expressed in B-cell malignancies, of which the chimeric anti-CD20 IgG1 mAb rituximab is the most widely used [24]. Rituximab, has proved to be a highly effective treatment for non-Hodgkin’s lymphoma and CLL.

An *in vitro* study by Dall’Ozzo et al. reported that the concentration of rituximab needed for 50% lysis (EC_50_) of a Burkitt lymphoma cell line by natural killer (NK) cells from healthy donors who had the *FCGR3A* 158 V/V genotype was 4.2 times lower than that observed with NK cells from donors who had *FCGR3A* 158 F/F [25]. However, this was only seen at very low concentrations of rituximab, much lower than those observed *in vivo* with normal dosing schedules, and a differential was not seen at higher concentrations [26,27]. Moreover, for a given concentration of a therapeutic mAb such as rituximab, the interplay between multiple factors may determine its anti-tumour activity *in vivo*. For example, at clinically relevant rituximab concentrations i*n vitro,* serum complement suppressed the induction of target cell death by ADCC and most markedly in effector cells that carried the 158 F/F genotype [28].

A beneficial effect of the *FCGR3A* 158 V/V genotype was reported by Cartron et al. in a study of 49 patients who had received rituximab for follicular lymphoma [29]. *FCGR3A* 158 V/V patients, who accounted for one fifth of the study population, had an improved response, with 100% and 90% objective response rates at 2 months and 12 months, respectively, compared with 2 month and 12 month response rates of 67% (*p* = 0.03) and 51% (*p* = 0.03) respectively, in *FCGR3A* 158 F carriers. However, there was no statistically significant difference in PFS although a trend was observed.

In a study of 87 patients with follicular lymphoma who had been treated with rituximab [30], *FCGR3A* 158 V/V patients also showed a higher response rate to rituximab treatment. This study found significant differences in PFS at 2 years: 45% for patients who were *FCGR3A* 158 V/V, and 14% for patient who were either *FCGR3A* 158 V/F or F/F (12% for V/F, 16% for F/F). Patients homozygous for the V allele of *FCGR3A* showed a higher response rate to rituximab treatment [30].

A retrospective study of patients with follicular lymphoma treated with rituximab in combination with chemotherapy found that overall survival was improved in patients with the *FCGR3A* 158 V/V genotype [31]. DNA from 142 patients was extracted from tissue preserved in paraffin blocks. It should be noted the *FCGR3A* genotype could not be determined in 22 patients. The authors concluded that patients with at least one *FCGR3A* V allele was associated with improved overall survival versus the F/F genotype (HR = 0.33, 95% CI, 0.11, 0.96, *p* = 0.042). Furthermore, for overall survival, there was evidence of a statistical interaction between the use of rituximab and the number of *FCGR3A* V alleles present (0, 1, or 2) (*p* = 0.006). Differences in the *FCGR2A* genotype were not found to correlate with outcome [31].

In patients with diffuse large B-cell lymphoma, Kim *et al.* reported that the *FCGR3A* 158 V allele was associated with a significantly higher complete response rate to a combination of rituximab, cyclophosphamide, doxorubicin and prednisolone [32].

Not all studies show a significant correlation between FcgR SNPs and clinical response to rituximab therapy. Dornan *et al.* assessed the progression free survival in a retrospective study of 419 CLL patients receiving fludarabine and cyclophosphamide or FC plus rituximab [33]. They concluded that *FCGR2A* and *FCGR3A* polymorphisms did not significantly influence response rate and PFS in either treatment arm.

The recent PRIMA study reported that *FCGR2A* and *FCGR3A* polymorphisms do not influence the response rate and outcome of follicular lymphoma patients treated with rituximab, either when it is combined with chemotherapy or used as maintenance treatment [26]. The analysis used peripheral blood DNA from 460 patients out of 1217 patients enrolled in this open-label, randomised clinical trial. Patients received one of three possible induction therapies containing rituximab in combination with a chemotherapy regimen. Following induction treatment; patients who achieved a complete response, or a partial response were randomly assigned (ratio 1:1) to observation or rituximab maintenance treatment. The authors reported complete responses after induction therapy in 65%, 67%, 66% (*p* = 0.86) of *FCGR3A* with the VV, VF, FF genotypes, and 60%, 73%, 66% (*p* = 0.21) of *FCGR2A* with the HH, HR, RR genotypes, respectively. After 2 years of maintenance therapy, response rates or PFS were not found to be influenced by FcgR genotype. However, the authors did report that FCGR3A polymorphisms were associated with the rate of grade 3–4 neutropenia during induction therapy consistent with previous reports that immune mechanisms mediated by NK cells may play a role in rituximab-induced neutropenia [34]. Two previous small retrospective studies of rituximab in follicular lymphoma patients also did not report any correlation between *FCGR2A* or *3A* polymorphisms and outcome [35,36].

In conclusion, although several small studies in lymphoma have shown that FcgR polymorphisms may be useful in predicting response to single agent rituximab [29,30,32] and have all shown the same favorable genotype, none of the analyses showed a statistically different PFS based on FcgR genotype. The larger studies examining the effects of FcgR polymorphisms on the outcome of follicular lymphoma and CLL patients treated with rituximab combined with chemotherapy showed no association between FcgR genotype and either response rate or outcome [26,33].

### Cetuximab

Cetuximab is a chimeric IgG1 mAb directed to the extracellular domain of the EGFR which is expressed at high levels in many epithelial tumours. It has been approved to treat *KRAS* wild-type metastatic colorectal cancer and head and neck cancer.

A retrospective study in metastatic colorectal cancer patients treated with cetuximab plus irinotecan found that patients with the homozygous *FCGR2A* 131 H/H and/or *FCGR3A* 158 V/V genotypes had a longer PFS than patients who carried a 131 R allele or a 158 F allele (5.5 months vs 3.0 months *p = 0.005*) [37]. The analysis included 69 patients who had all received and progressed through one irinotecan-containing chemotherapy regimen. The majority of patients had actually received two-lines of prior therapy. Bibeau et al. concluded that when combined, *FCGR2A*/*FCGR3A* genotypes are prognostic factors for disease progression in metastatic colorectal cancer patients treated with the combination of cetuximab and irinotecan [37]. A reservation about these data is that there is deviation from the Hardy-Weinberg equilibrium indicative of methodological problems (Table 2).

In a study of 106 patients with metastatic colorectal cancer who had been treated with cetuximab and standard chemotherapy (only defined as irinotecan or oxaliplatin-based), Rodriguez et al. reported that patients with any *FCGR2A* 131H and/or *FCGR3A* 158 V allele were more likely to show a response or have stable disease (65% vs 35% for other genotypes; *p* = 0.014). The study recruitment occurred prior to the routine pre-selection of patients for cetuximab treatment on the basis of *KRAS* mutation status. The authors state that the presence of *KRAS* mutations only accounted for 30-40% of patients who do not respond to cetuximab. Furthermore, they summarised literature that reported patients with *KRAS* mutant tumours who still responded to cetuximab [38]. On this basis, they wanted to determine if FcgR genotype would predict which patients with a *KRAS*, or other downstream mutations, would respond to cetuximab. Thus, only 44 patients who were later found to have either a *KRAS*, *BRAF*, *NRAS* or *PI3K* mutation were included in the FcgR genotype analysis [39]. Somewhat surprisingly, these selection criteria resulted in patients whose tumours were *KRAS* wild type and thus who might reasonably be expected to respond to cetuximab [40] being excluded from the analysis.

Etienne-Grimaldi et al. examined the effect of *FCGR2A* H131R and *FCGR3A* V158F SNPs on the efficacy of cetuximab in metastatic colorectal cancer patients treated with cetuximab and irinotecan in combination with oral tegafur-uracil [41]. Germline DNA samples from 52 patients were available for FcgR genotyping. A non-significant trend towards a better response rate was observed in *FCGR3A* 158 V-carriers (62.1% in V/V or V/F patients vs 26.3% in F/F; *p* = 0.020, where a multiple comparisons test set the level of statistical significance at less than or equal to 0.010). A longer, although non-statistically significant, overall survival was also reported in *FCGR3A* 158 V-carriers (20.9 months for V/V or V/F patients vs 12.4 months for F/F; *p* = 0.032).

In contrast, a study in metastatic colorectal cancer patients treated with cetuximab found that *FCGR3A* 158 V/V patients had a shorter PFS compared with 158 V/F or F/F patients [42]. The analysis included 39 patients who were part of the phase II open-label multicenter study (ImClone trial 0144) of cetuximab. Genotyping was performed on genomic DNA extracted from peripheral blood. A combined analysis of the *FCGR2A* 131 and *FCGR3A* 158 polymorphisms showed that patients with the favorable genotypes found in this study (*FCGR2A*, any H allele, and *FCGR3A*, any F allele) showed a median PFS of 3.7 months (95% CI, 2.4 to 4.4 months), whereas patients with any two unfavorable genotypes (*FCGR2A* 131 R/R or *FCGR3A* 158 V/V) had a PFS of 1.1 months (95% CI, 1.0 to 1.4 months; *p* = 0.004 )[42]. The results of this study have been supported by a randomised phase II clinical trial examining the use of cetuximab in combination with bevacizumab in irinotecan-refractory colorectal cancer patients (the BOND-2 study) [43]. It showed that *FCGR3A* 158 F/F patients had a significantly better response to the combination therapy compared to patients who were F/V or V/V (n = 31, response rates of 56%, 25% and 8% respectively). However, there are some concerns regarding the significant deviation from Hardy-Weinberg equilibrium in this study (Table 2).

More recently a diminished survival for patients with the *FCGR3A* 158 V/V genotype (as compared to V/F or F/F) has also been demonstrated by Dahan et al. in a study of 58 patients with advanced colorectal cancer who had received irinotecan in combination with cetuximab [44]. This effect was present in the entire study population and in a sub-analysis of patients with KRAS wild type tumours. The authors concluded that the *FCGR3A* V158F polymorphism is a significant independent predictor of overall survival.

However, a retrospective analysis published by Paez et al. concluded that FcgR genotype was not useful in predicting response to cetuximab or panitumumab (an anti-EGFR mAb) in patients with advanced colorectal cancer [45]. The study included 104 patients with metastatic colorectal cancer who were treated with either cetuximab or panitumumab after progressing through at least one prior chemotherapy regimen. Overall, 92 patients (88%) were treated with cetuximab plus chemotherapy and 12 patients (12%) were treated with panitumumab alone or in combination with irinotecan. No significant difference was observed for tumor response and PFS between *FCGR2A* or *FCGR3A* genotype subsets. This remained the case regardless of *KRAS* status. The authors concluded that the only reliable biomarkers to predict response to anti-EGFR therapies are *KRAS* status and the presence of skin toxicity.

In summary, the data to support the use of *FCGR2A* and *FCGR3A* polymorphisms to predict the response to cetuximab are inconsistent. Three retrospective studies report that the V/V genotype is the most beneficial FCGR3A genotype [37,39,41], whereas three other retrospective studies report that the F/F genotype is the most beneficial [42-44]. The inconsistent findings suggest that FcgR polymorphisms are not currently useful predictive biomarkers of response to cetuximab.

### Methodological considerations for FcgR genotyping

The inconsistent relationship between *FCGR3A* genotype and indicators of clinical benefit precludes the use of FcgR polymorphisms as predictive markers of response to monoclonal antibodies in cancer patients. Although the reason for these discordant findings remains unclear, methodological factors may be of critical importance. Differences in the tissues used or flaws in methodology may account for some of the discrepancies between the various clinical studies. Methodological flaws may sometimes be responsible when significant deviations from the Hardy-Weinberg equilibrium are seen. This section will briefly review the issues relevant to FcgR genotyping studies.

The source of tissue for FcgR genotyping is an important consideration. Ideally, normal tissue should be used for genotyping as the germline genotype determines the phenotype of the cytotoxic T-cells which mediate ADCC. However, in some studies, only surgically removed tumour material will be available. Tumour DNA may have undergone allelic loss (also known as loss of heterozygosity: LOH) and thus may not enable accurate genotyping in some cases. Adjacent normal tissue can often be insufficient for DNA extraction and may not be truly normal. Attempting to genotype using formalin fixed paraffin embedded (FFPE) samples adds another level of complexity due to the fragmentation and degradation of the DNA.

However, even if good quality DNA from peripheral blood is used, accurate genotyping of the *FCGR3A* V58F polymorphism is methodologically challenging because of the very high degree of sequence homology between *FCGR3A* and *FCGR3B*. These two genes are more than 99% homologous in the region flanking the V158F polymorphism thus placing constraints on the design of *FCGR3A* -specific PCR primers. To amplify *FCGR3A* preferentially and to minimize amplification of *FCGR3B*, it is necessary to discriminate against *FCGR3B* by placing the 3’ end of the primer at one of the very few places where the sequence of *FCGR3B* is different from *FCGR3A*. (In several reports, a nested PCR approach was used. In our opinion, nested PCRs should be used with caution because of the very real danger of inadvertent PCR contamination.)

Methods that do not sufficiently discriminate against the pseudogene which has a G at the position corresponding to the rs396991 SNP will lead to an overcalling of heterozygotes and a corresponding undercalling of TT homozygotes. Hardy-Weinberg equilibrium analysis may be used to determine whether this might be occurring for a given methodology. Deviation from the Hardy-Weinberg equilibrium may also occur in large multicentre studies where diverse ethnic groups are represented but in this case, a preponderance of homozygotes might be expected if the populations differ in allelic frequency. Table 2 shows the fit with Hardy-Weinberg equilibrium in the studies where individual genotype frequencies are reported and indicates that several deviate from expectations.

The opportunities for PCR primer design are limited further if an FFPE tumour sample is used because the highly fragmented DNA obtained from FFPE makes it necessary to design short amplicons flanking the diagnostic SNP. In the case of *FCGR3A*, if only (FFPE) tumour tissue is available, the best methodology is one that is sensitive to the presence of both alleles, even if one is present at low frequency due to LOH. To favour specific amplification from *FCGR3A*, we have recently adopted a Pyrosequencing approach as this allows the determination of allelic frequency even when one of the alleles is present at less than 10% (Mellor, Mikeska and Dobrovic; manuscript in preparation).

## Conclusions

The functional FcgR polymorphisms have been reported as novel pharmacogenetic biomarkers that could be used to better target the use of mAbs in cancer patients. However, the studies that we have reviewed do not describe a consistent effect of FcgR genotype on the clinical anti-tumour activity of therapeutic mAbs of IgG1 isotype. The inconsistencies found in these studies include the tumour type, the cytotoxic agents used in combination, the clinical setting (metastatic vs adjuvant for trastuzumab), the clinical benefit parameters measured, the therapeutic antibody used, as well as the magnitude and even the direction of the effect. Even when there was agreement between studies about the most beneficial genotype, subgroups of patients with less favorable genotypes still appeared to derive some treatment benefit from mAb therapy.

Many of the studies presented here were retrospective and non-randomised and cannot adequately determine the nature of the relationship of host FcgR genotype to the anti-tumour activity of the mAbs. Significantly, several well-powered studies did not support the hypothesis that FcgR genotype predicts the therapeutic effect of these agents.

Although FcgR genotype may be a factor contributing to both the anti-tumour activity and clinical benefit of these therapeutic mAbs, other factors may also be important.

(i) The *FCGR2A* H131R and *FCGR3A* V158F polymorphisms are in linkage disequilibrium and their contributions may be difficult to disentangle [46,47].

(ii) Direct cytotoxicity of the mAbs, which depends on Fab binding rather than Fc binding and which is enhanced by the chemo- and radio-sensitizing properties of these mAbs;

(iii) Predominance of non-ADCC mechanisms of action, including complement-dependent cytotoxicity, apoptosis and phagocytosis

(iv) Pharmacokinetic properties of the mAbs such as the maximum concentration achieved in relation to antigenic mass [48];

(v) Fab-mediated tumour cell cytotoxicity and FcgR-mediated endocytosis and phagocytosis of tumour antigens that together initiate cellular immunity, which may be slow in onset and durable [12,49];

Standardising methodologies for accurate genotyping is required before definitive conclusions can be drawn. We also need to await the outcomes of more large trials using robust testing methodologies before we can reach a definitive conclusion about the predictive utility of FcgR polymorphisms. However, even a significant difference in tumour response based on FcgR genotype cannot reasonably be used to exclude patients from mAb therapy if the distinct possibility of a tumour response remains. Hence, we consider that it is currently not clinically appropriate to deny mAb therapies to patients on the basis of their FcgR genotype.

## Abbreviations

ADCC: Antibody-dependent cellular cytotoxicity; CLL: Chronic lymphocytic leukemia; DFS: Disease free survival; FcgR: Fc gamma receptor; FCGR2A: Fc gamma receptor 2A gene; FCGR3A: Fc gamma receptor 3A gene; FFPE: Formalin-fixed paraffin embedded; HER2: Human epidermal growth factor receptor; IgG1: Immunoglobulin IgG1; LOH: Loss of heterozygosity; mAb: Monoclonal antibody; NK: Natural killer (cell); PFS: Progression free survival; SNP: Single nucleotide polymorphism.

## Competing interests

The authors have no competing interests to declare in relation to this manuscript.

## Authors’ contributions

JDM, HRI, JRZ and AD were responsible for the conception and design of the manuscript. JDM, MPB, HRI, JRZ and AD participated in drafting, review and/or revision of the manuscript. All authors read and approved the final submitted manuscript.
